# A Rare Autoimmune Triad: Coexistence of Systemic Lupus Erythematosus, Sjögren’s Syndrome, and Ankylosing Spondylitis: A Case Report

**DOI:** 10.1155/crrh/2532559

**Published:** 2026-10-07

**Authors:** Gianna Sagona, Frank Schwartz, Molly Nguyen, Saad Tariq

**Affiliations:** ^1^ University of Illinois College of Medicine Rockford Rockford Illinois USA; ^2^ Department of Rheumatology University of Illinois College of Medicine Rockford Illinois USA uic.edu

**Keywords:** ankylosing spondylitis, case report, Sjögren’s syndrome, systemic lupus erythematosus, upadacitinib

## Abstract

Systemic lupus erythematosus (SLE), Sjögren’s syndrome (SS), and ankylosing spondylitis (AS) are distinct immune‐mediated diseases with different classification frameworks and usual treatment approaches. Coexistence of all three disorders in a single patient is rare and creates diagnostic and therapeutic challenges. We report a 40‐year‐old woman with longstanding SLE, SS associated with SLE, and subsequently recognized axial spondyloarthritis (axSpA)/AS. The patient fulfilled the 2019 ACR/EULAR classification criteria for SLE (total score ≥ 10), the 2016 ACR/EULAR classification criteria for primary SS (total score ≥ 4), and the 2009 ASAS classification criteria for axSpA through both the imaging and clinical arms. Conventional treatment options were limited by prior gastrointestinal bleeding with NSAIDs and concern for drug‐induced lupus‐like syndromes with TNF inhibitors. The patient remained stable on hydroxychloroquine 200 mg daily and was enrolled in a Phase 2 SLE clinical trial evaluating a JAK inhibitor. Treatment assignment was unknown during the blinded period; during the unblinded continuation phase, she was confirmed to be receiving upadacitinib 15 mg daily. Over a 6‐month duration of confirmed upadacitinib exposure, clinical improvement in lupus‐ and sicca‐related symptoms were observed, and follow‐up MRI showed resolution of previously documented sacroiliitis. Although causality cannot be established from a single uncontrolled observation, the clinical and imaging improvement during confirmed upadacitinib exposure is hypothesis‐generating and suggests that shared inflammatory pathways may warrant further investigation in selected overlap cases.

## 1. Introduction

Systemic lupus erythematosus (SLE), Sjögren’s syndrome (SS), and ankylosing spondylitis (AS) are clinically and immunologically distinct disorders. SLE is a multisystem autoimmune disease characterized by antinuclear antibodies (ANAs) and immune complex–mediated inflammation, with classification supported by the 2019 EULAR/ACR criteria [1]. SS is characterized by autoimmune involvement of lacrimal and salivary glands, often presenting with keratoconjunctivitis sicca and xerostomia, and may occur alone or in association with another systemic autoimmune disease; the 2016 ACR/EULAR classification criteria provide a validated framework for diagnosis based on a weighted scoring system [2]. AS belongs to the axial spondyloarthritis (axSpA) spectrum and is characterized by inflammatory back pain, sacroiliitis, and a strong association with HLA‐B27; the 2009 ASAS classification criteria define axSpA through an imaging arm (sacroiliitis on MRI or radiograph plus ≥ 1 SpA feature) or a clinical arm (HLA‐B27 plus ≥ 2 SpA features) [3]. Although autoimmune diseases may coexist, overlap between SLE and SS is well recognized, whereas concurrent AS in a single patient is exceptionally unusual. Such cases can be difficult to interpret because musculoskeletal symptoms may be attributed to lupus arthritis, degenerative disease, or fibromyalgia unless inflammatory axial disease is specifically considered. Treatment decisions are also complex. NSAIDs and biologic agents, including TNF and IL‐17 inhibitors, are standard therapies for AS, whereas SLE is commonly treated with hydroxychloroquine, corticosteroids, immunosuppressant, and selected biologics [1, 3]. In patients with active lupus, TNF inhibitors may be clinically challenging due to association with drug‐induced lupus‐like syndromes [4].

We report a rare case of coexisting SLE, SS associated with SLE, and HLA‐B27‐positive axSpA/AS with MRI‐confirmed bilateral sacroiliitis. The patient was enrolled in a lupus clinical trial evaluating upadacitinib; treatment assignment was blinded during the initial study period, and she was subsequently confirmed to have received upadacitinib 15 mg daily during the open label extension. Upadacitinib is approved for the treatment of AS and has shown efficacy in a Phase 2 clinical trial for SLE [5, 6]. While causality cannot be established from a single case and her long‐term outcomes remain unknown, the patient’s clinical and imaging improvement during confirmed upadacitinib exposure is presented as a hypothesis‐generating observation that warrants further investigation into shared inflammatory pathways and potential therapeutic targets in autoimmune–autoinflammatory overlap syndromes.

## 2. Report of the Case

A 40‐year‐old woman with a longstanding history of SLE and SS associated with SLE presented with Raynaud’s phenomenon, malar rash, subacute cutaneous lupus, nonscarring alopecia, history of mucosal ulcers, objective inflammatory arthritis, and chronic sicca symptoms.

### 2.1. SLE Classification

The patient fulfilled the 2019 ACR/EULAR classification criteria for SLE with a total score of 12, based on a positive ANA entry criterion and mucocutaneous and musculoskeletal manifestations [1].

### 2.2. SS Classification

The patient fulfilled the 2016 ACR/EULAR classification criteria for SS with a score of at least 4 points [7]. Her score was based on positive anti‐SSA/Ro antibodies and objective ophthalmologic confirmation of keratoconjunctivitis sicca, supported by chronic ocular and sicca symptoms for more than 6 months [2].

### 2.3. axSpA Classification

The patient reported chronic inflammatory low back pain of greater than 3 months’ duration with onset before age 45, prolonged morning stiffness, peripheral inflammatory arthritis, a history of colitis, HLA‐B27 positivity, and first‐degree family history of AS. Although radiographs of the sacroiliac joints did not demonstrate definite sacroiliitis, MRI of the pelvis performed in February 2025 demonstrated active bilateral sacroiliitis with subchondral bone marrow edema, meeting the ASAS definition of a positive MRI for sacroiliitis ([7], Figure 1a). Based on the overall clinical presentation, serologic findings, and imaging, the patient fulfilled the 2009 ASAS classification criteria for axSpA through both the imaging and clinical arms and was diagnosed with AS by her treating rheumatologist [3]. The imaging arm was satisfied by MRI‐confirmed active sacroiliitis plus at least one additional spondyloarthritis feature; this patient had multiple qualifying features, including inflammatory back pain, peripheral arthritis, inflammatory bowel disease, HLA‐B27 positivity, and a first‐degree family history of AS. The clinical arm was independently fulfilled by HLA‐B27 positivity in combination with at least two additional spondyloarthritis features as noted above in this patient [3].

**FIGURE 1:**
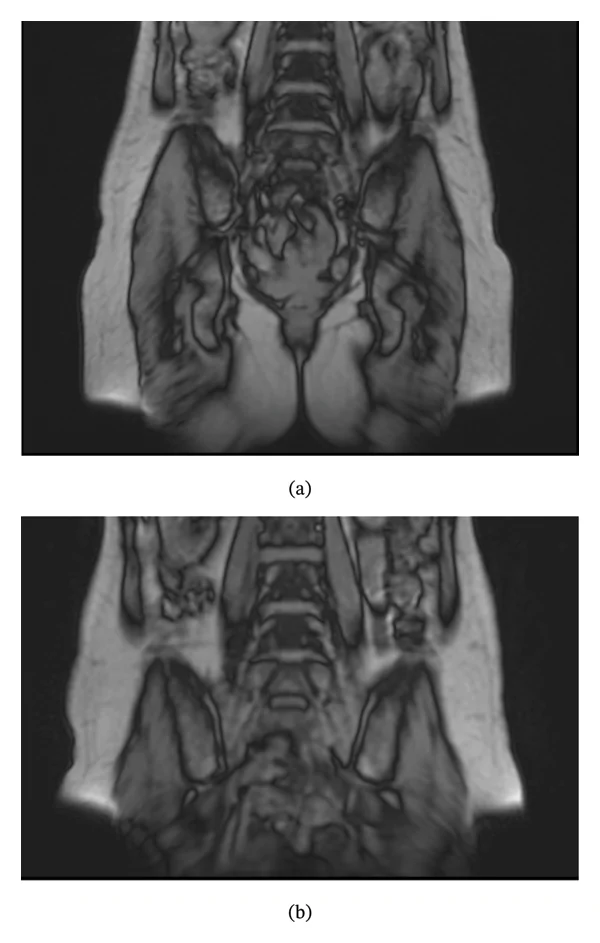
(a) MRI of the pelvis without and with contrast, February 2025. Active bilateral sacroiliitis, more pronounced on the right, with fluid signal at the posterior inferior aspect of the sacroiliac joints (red arrows). Mottled marrow signals of iliac bones and sacrum. (b) MRI of the pelvis without and with contrast, April 2025, showing resolution of the previously documented sacroiliitis (blue arrows). No abnormal enhancement. Normal bone marrow signal intensity.

### 2.4. Laboratory Findings

Her history was negative for thrombosis, cytopenia, or other antiphospholipid manifestations. Laboratory studies consistently demonstrated positive ANA with dual patterns (homogenous at > 1:1280 and speckled at 1:640). High‐titer anti‐SSA/Ro antibodies were positive, while anti‐dsDNA, anti‐Smith, and antiphospholipid antibodies were negative. Complements (C3 and C4) remained stable within the normal range.

### 2.5. Treatment

Prior to enrollment in the clinical trial, the patient was maintained on hydroxychloroquine 200 mg daily for SLE and pilocarpine for management of Sjögren’s‐related sicca symptoms. Previous therapeutic approaches included NSAIDs, which were discontinued due to a history of gastrointestinal bleeding, as well as intra‐articular corticosteroid injections for symptomatic management of inflammatory arthritis. Due to persistent disease activity, including inflammatory arthritis, active sacroiliitis, and ongoing SLE manifestations despite conventional therapy, the patient was enrolled in a Phase 2 clinical trial evaluating the Janus kinase (JAK) inhibitor upadacitinib for SLE (5). Treatment assignment was blinded during the initial study period; the patient and treating physicians did not know whether she received active drug or placebo. Following completion of the blinded phase, she entered the open‐label extension and was confirmed to have received upadacitinib 15 mg daily throughout. After more than 6 months of confirmed upadacitinib exposure, the patient demonstrated clinical improvement in lupus‐ and sicca‐related symptoms, and follow‐up MRI of the pelvis performed in April 2025 showed resolution of previously documented bilateral sacroiliitis (Figure 1b).

## 3. Discussion

SLE is a systemic autoimmune disease characterized by autoantibody production and immune complex–mediated inflammation affecting the skin, joints, kidneys, hematologic, and nervous systems. The 2019 EULAR/ACR classification criteria have a sensitivity of 96.1% and a specificity of 93.4% [1]. Current treatment includes hydroxychloroquine as a cornerstone, corticosteroids, immunosuppressants, and FDA‐approved biologics including belimumab and anifrolumab. The present patient fulfilled the 2019 ACR/EULAR criteria with a score of 12, driven by mucocutaneous and musculoskeletal manifestations [1].

### 3.1. SS

SS can occur either as a primary disease or in association with other autoimmune disorders, most commonly SLE or rheumatoid arthritis. It is characterized by xerostomia and keratoconjunctivitis sicca, though systemic features such as arthritis, Raynaud’s phenomenon, cytopenias, or visceral involvement may occur. Autoantibodies include ANA, anti‐SSA/Ro, and anti‐SSB/La, which overlap with SLE serology. Treatment remains largely symptomatic, with systemic therapy reserved for severe extra glandular disease; no therapies have formal regulatory approval for SS, though biologics and JAK inhibitors are under active investigation [8]. Preclinical and translational studies have demonstrated activated JAK‐STAT signaling in salivary gland tissue from SS patients, with JAK inhibition normalizing interferon‐driven immune activation at the tissue level [9]. The present patient had clinical SS associated with SLE, noted by chronic sicca symptoms including keratoconjunctivitis sicca and xerostomia for more than 6 months. She fulfilled the 2016 ACR/EULAR SS classifications criteria with positive anti‐SSA antibodies and objective confirmation of keratoconjunctivitis sicca on ophthalmologic examination, for a score of at least 4 points [2].

AS belongs to the axSpA spectrum and is characterized by inflammation of the sacroiliac joints and axial skeleton, with a strong association with HLA‐B27. The 2009 ASAS classification criteria define axSpA through an imaging arm (sacroiliitis on MRI or radiograph plus ≥ 1 SpA feature) and a clinical arm (HLA‐B27 plus ≥ 2 SpA features) [3]. MRI detects early inflammatory lesions, particularly subchondral bone marrow edema, whereas radiographs reveal later structural changes such as sclerosis and syndesmophytes [10]. Management includes NSAIDS, physical therapy, and advanced therapies including TNF inhibitors, IL‐17 inhibitors, and JAK inhibitors [7]. Upadacitinib, a selective JAK1 inhibitor, demonstrated efficacy in the SELECT‐AXIS 1 trial, achieving ASAS40 response in 52% vs. 26% with placebo at week 14 in patients with active AS [6], and its efficacy in nonradiographic axSpA was subsequently confirmed in the Phase 3 SELECT‐AXIS 2 trial, supporting regulatory approval across the axSpA spectrum [11]. The present patient fulfilled the ASA criteria through both arms.

### 3.2. Rarity of Overlap Syndromes

Autoimmune diseases frequently coexist, with the strongest associations occurring among connective tissue diseases, particularly SS, SLE, and systemic sclerosis [12]. The coexistence of SLE and SS in this patient is, therefore, consistent with well‐established epidemiological patterns. However, AS is not identified among the commonly co‐occurring diseases, suggesting the addition of AS to this combination is exceptionally uncommon. Previous research has shown that SLE is associated with at least one additional autoimmune disease in 41% of the patients, most commonly autoimmune thyroid disease, antiphospholipid syndrome, and SS—all HLA class II‐associated conditions—whereas spondyloarthritis was not among the co‐occurring diseases reported [13]. According to the autoimmune–autoinflammatory continuum model, SLE and SS lie at the autoimmune end of the spectrum, driven by adaptive immunity, autoantibody production, and Type I interferon signaling, whereas AS occupies a mixed, predominantly autoinflammatory pattern of IL‐17/IL‐23‐driven inflammation and HLA‐B27‐mediated innate immune activation [14]. Consequently, the coexistence of SLE and AS has been regarded as an immunologic paradox because these diseases arise through fundamentally different pathogen pathways. Similar paradoxical overlap syndromes have been described between SLE and sarcoidosis, despite their divergent immunologic mechanisms, further demonstrating that diseases traditionally viewed as immunologically distinct can rarely coexist [15]. Reports of similar paradoxical overlap syndromes are rare, prompting a systematic literature review to identify previously published cases describing the coexistence of SLE, SS, and AS in a single patient. The search included PubMed/MEDLINE, Google Scholar, OpenEvidence, and University of Illinois College of Medicine Rockford library databases. Search terms included combinations of “systemic lupus erythematosus,” “Sjögren’s syndrome,” “ankylosing spondylitis,” “spondyloarthritis,” “overlap syndrome,” and “coexistence,” with no restrictions on language or publication date. Reference lists of relevant articles were also reviewed for any additional reports. The search was last updated on 7/20/2026. Only one comparable case was identified: a Bangladeshi woman with RA, SLE, SS, AS, and antiphospholipid syndrome who died from rupture of ascending aortic aneurysm [16]. No other published cases describing the coexistence of SLE, SS, and AS in a single patient were identified, highlighting the extreme rarity of this autoimmune overlap.

### 3.3. Therapeutic Implications of JAK Inhibition

This patient did not tolerate or respond well to conventional therapy. TNF inhibitors, although highly effective in AS, were avoided because of her lupus. She was, therefore, enrolled in a lupus clinical trial and initiated on a JAK inhibitor (upadacitinib 15 mg daily). Although long‐term outcomes remain unknown, the JAK/STAT pathway plays a central role in cytokine signaling across SLE, SS, and AS. Clinical evidence supports JAK inhibition in SLE, early trials and translational data suggest potential benefit in SS, and randomized clinical trials confirm efficacy in AS [5, 6, 9, 17, 18]. Notably, in the Phase 2 SLEek trial, efficacy in SLE was demonstrated for upadacitinib 30 mg daily, whereas the present patient received the AS‐approved dose of 15 mg daily; this distinction reinforces that her response is hypothesis‐generating rather than confirmatory [5].

### 3.4. Limitations and Future Directions

Several limitations should be acknowledged. This is a single case report, and conclusions cannot be generalized. The clinical improvement was observed during an open‐label extension phase, and the absence of concurrent control limits causal inference. Formal disease activity indices were not systematically collected outside the clinical trial protocol. The follow‐up MRI was performed approximately 2 months after the baseline study, and longer‐term imaging follow‐up will be needed to confirm durability of the response. Additionally, upadacitinib is FDAapproved for active AS and nonradiographic axSpA, but its use in SLE remains investigational; the Phase 2 SLEek trial showed promising results at the 30‐mg dose [5].

## 4. Conclusion

The coexistence of SLE, SS associated with SLE, and HLA‐B27‐positive axSpA/AS in a single patient is exceptionally rare. This case underscores the importance of recognizing autoimmune overlap syndromes and considering shared pathogenic mechanisms when selecting therapy. Future prospective studies of patients with overlapping autoimmune disease could help determine whether JAK inhibitors offer a meaningful therapeutic advantage in this population.

## Author Contributions

Gianna Sagona: conceptualization (supporting), writing–originating draft (lead), and writing–review and editing (equal). Frank Schwartz: writing–review and editing (equal). Molly Nguyen: writing–review and editing (equal). Saad Tariq: conceptualization (lead), writing–original draft (supporting), and writing–review and editing (equal).

## Funding

The authors have nothing to report.

## Ethics Statement

Written informed consent was obtained from the patient for publication of this report and accompanying images.

## Conflicts of Interest

The authors declare no conflicts of interest.

## Supporting Information

Additional supporting information can be found online in the Supporting Information section.

## Supporting information


**Supporting Information** 082926_CAREchecklist.

## Data Availability

The data that support the findings of this study are available on request from the corresponding author. The data are not publicly available due to privacy or ethical restrictions.
